# MCPA Optical Fiber Sensors via Molecularly Imprinted Polymers Combined with Intensity-Based and Plasmonic Platforms

**DOI:** 10.3390/polym17223048

**Published:** 2025-11-17

**Authors:** Ines Tavoletta, Francesco Arcadio, Luigi Zeni, Ricardo Oliveira, Rogério Nunes Nogueira, Giancarla Alberti, Nunzio Cennamo

**Affiliations:** 1 Department of Engineering, University of Campania Luigi Vanvitelli, Via Roma 29, 81031 Aversa, Italyfrancesco.arcadio@unicampania.it (F.A.);; 2 Instituto de Telecomunicações, University of Aveiro, Campus Universitário de Santiago, 3810-193 Aveiro, Portugal; 3 Department of Chemistry, University of Pavia, Via Taramelli 12, 27100 Pavia, Italy

**Keywords:** optical–chemical sensors, intensity-based sensors, plasmonic sensors, plastic optical fibers (POFs), molecularly imprinted polymers (MIPs), 4-chloro-2-methylphenoxyacetic acid (MCPA)

## Abstract

Two low-cost optical–chemical sensors based on plastic optical fibers (POFs) and molecularly imprinted polymers (MIPs) are developed and tested for the detection of 4-chloro-2-methylphenoxyacetic acid (MCPA), a herbicide of great interest in environmental monitoring. The first sensor is based on an optical splitter composed of two modified POFs coupled with an MIP for measuring MCPA. The second type of sensor is based on a surface plasmon resonance (SPR) D-shaped POF platform combined with the same MIP receptor for MCPA. The two proposed polymer-based sensors, exploiting different optical phenomena, were tested using similar equipment, consisting of white light sources and spectrometers. The experimental results show that both MCPA sensors present high selectivity for the target analyte and similar performances in terms of detection limits (LODs) of 3 nM and detection ranges (between 3 nM and 500 nM) by exploiting the MIP’s sites with a similar affinity constant. The polymer-based sensors exhibited better performances than those achieved by the electrochemical technique combined with the same MIP presented in the literature. Then, tests performed on real samples demonstrated good recovery values (between 82% and 116%), assessing the applicability of both sensors in real-world scenarios. Moreover, the POF-MIP splitter sensor configuration can be fabricated without expensive fabrication steps, such as spinning and sputtering processes.

## 1. Introduction

Sensing techniques have emerged as a cornerstone of technological advancement, particularly when combined with state-of-the-art microfabrication approaches. These methods enable the development of compact, highly sensitive, and specific sensors, which find applications across various fields, including environmental monitoring, healthcare, industry, and agriculture. By leveraging the precision and miniaturization afforded by microfabrication, sensors, combined with specific molecular recognition elements (MREs), can detect analytes at extremely low concentrations, opening new possibilities for monitoring and managing critical environmental parameters [1,2,3,4,5,6]. In this context, molecularly imprinted polymers (MIPs) are the most promising technologies for detecting specific molecules. MIPs are synthetic receptors designed with highly selective recognition sites, created through polymerization around a molecular “template”. After removing the template, MIPs retain a chemical memory of the target molecule, allowing them to interact with it specifically [7,8]. This feature makes MIPs particularly well-suited for environmental applications, enabling the development of highly sensitive and specific chemical sensors, even in complex matrices [9,10]. Chemical sensors based on MIPs are notable for their capability to combine the great selectivity of molecular recognition sites with the chemical stability and robustness of polymeric materials, which distinguish MIPs and make them advantageous over biological receptors, such as antibodies [7,8,9,10]. When combined with Internet of Things (IoT) technologies, these sensors become even more impactful, enabling large-scale, real-time environmental data collection and analysis. These solutions not only improve the ability to detect environmental contaminants promptly but also support the sustainable management of natural resources and the protection of ecosystems [11,12]. Chemical sensors can explore different types of transducers, such as optical or electrochemical technologies, to convert the MIP–analyte interaction into a detectable signal [10,13]. Optical sensors offer several advantages over electrochemical sensors, including higher sensitivity, allowing the monitoring of very low analyte concentrations, and the ability to operate in complex environments without being affected by chemical or electrolytic conditions. Additionally, optical sensors provide greater long-term stability as they do not suffer from electrode degradation and offer extensive possibilities for miniaturization and integration into portable or IoT devices. Lastly, they can be used remotely, making them ideal for distributed or hard-to-reach applications [13,14]. Among optical sensors, the most well-known ones exploit plasmonic phenomena, such as surface plasmon resonance (SPR). These offer high sensitivity and specificity by detecting variations in the refractive index (RI) at the sensor’s surface. In particular, sensors based on plastic optical fibers (POFs) and exploiting plasmonic phenomena represent an emerging technology with interesting advantages in terms of flexibility, affordability, and ease of use [15,16,17,18,19,20,21]. However, there is growing interest in intensity-based sensors that show performance comparable to plasmonic sensors [22,23]. These are based on variations in light intensity and can provide highly sensitive and accurate results while often being simpler in design and operation than plasmonic sensors. Due to their versatility, they find applications in fields such as environmental monitoring [24]. In this context, one of the most concerning environmental contaminants, herbicides, occupies a central position due to their widespread use and potential impacts on ecosystems [25,26,27]. Glyphosate (GLY), one of the most extensively used herbicides in the world, has drawn substantial attention because of its potential implications for human health and the environment. While highly effective in controlling weeds, GLY’s persistence in soil and water raises significant concerns [28,29]. Another noteworthy herbicide example is 4-chloro-2-methylphenoxyacetic acid (MCPA). This is a member of the phenoxyacetic acid family, commonly used to manage broadleaf weeds. MCPA has become a molecule of interest for environmental monitoring, given its extensive use and the risk of contaminating water resources [30]. Traditionally, the detection of pesticides and herbicides in environmental samples has relied on high-precision analytical methods, for example, gas chromatography (GC) and high-performance liquid chromatography (HPLC), often combined with mass spectrometry (GC-MS, LC-MS) or electrophoretic methods [31]. These conventional approaches offer high sensitivity and selectivity but also have significant drawbacks: they require complex sampling and time-consuming sample preparation procedures, such as extraction, pre-concentration, and purification steps, which are costly and unsuited for real-time or on-site monitoring [32].

Optical–chemical sensors and optical biosensors have emerged as promising alternatives to the gold-standard techniques for real-time and on-site environmental monitoring due to their low cost, small size, portable equipment, and rapid response times [33]. The integration of MIPs into optical sensing platforms improves the Technology Readiness Levels (TRLs) of the sensor systems, enabling the development of new devices for real-world applications [19,34]. However, many current optical sensors for environmental pollutants, such as those based on fluorescent probes or complex sensing architectures, face several limitations in terms of practical applicability in real-world scenarios. Moreover, some of these sensors exhibit long-term stability issues [24,35,36]. Recently, Zanoni et al. developed a potentiometric cell for the detection of phenoxy herbicides based on the use of an MCPA-specific MIP, achieving a detection limit of about 13 nM [37]. The combination of MIPs with the microfabrication technologies offers an innovative sensing approach to detecting molecules of interest [38,39,40]. In more detail, several works have been presented in the literature by our research group to compare the performances of microfabricated POF sensors (intensity-based sensors) with those of conventional plasmonic sensors based on POFs and MIPs [22,23].

In this work, two optical–chemical sensors are realized and tested for MCPA detection in water via MIPs and modified POFs. Specifically, a conventional SPR-POF-MIP sensor and a POF-based splitter configuration have been obtained by exploiting an MIP, previously synthesized for the development of an electrochemical sensor for phenoxy herbicides [37], to monitor the MIP-MCPA interactions at a nanomolar level, and demonstrating that although the MIP polymer is the same, by changing the transduction method, it is possible to obtain sensors with greater sensitivity. The specificity and selectivity of these POF-MIP sensors were demonstrated by comparing the results with those of the non-imprinted polymer (NIP) configurations and testing the MIP-based chemical sensors with interfering molecules. Finally, samples of real matrices have been tested to demonstrate the applicability of the sensor systems in a real scenario.

## 2. Materials and Methods

### 2.1. Reagents

4-chloro-2-methylphenoxyacetic acid (the template, MCPA), bentazon (BTZ), glyphosate (GLY), toluene, absolute ethanol, and 2,2-azobisisobutyronitrile (the radical initiator, AIBN) were used as received from Merk Life Science S.r.l. (Milan, Italy). Methacrylic acid (the functional monomer, MAA) and ethylene glycol di-methacrylate (98%, crosslinking reagent, EGDMA) are from Merk Life Science S.r.l. (Milan, Italy). Before use, they were purified by solid-phase extraction (SPE) with a cartridge packed with aluminum oxide (Carlo Erba, Milan, Italy) to remove stabilizers. All solutions were prepared with ultrapure water, following a previously described protocol by Zanoni et al. [37].

### 2.2. Molecularly Imprinted Polymer (MIP) and Non-Imprinted Polymer (NIP) Prepolymeric Mixture Preparation

The prepolymeric mixture was prepared by adapting the previously proposed procedure described in the paper by Zanoni et al. [37], in which the MIP was deposited onto the working electrode surface of a screen-printed electrochemical cell to prepare a potentiometric sensor for phenoxy herbicides. The procedure is here briefly summarized. The mixture was prepared in a laminar flow hood. A total of 56 mg of the template compound MCPA (0.276 mmol) was dissolved in 0.4 mL of toluene. Subsequently, 0.35 mL of the functional monomer MAA (4.14 mmol), 0.35 mL of the crosslinking agent EGDMA (1.86 mmol), and 50 mg of the initiator AIBN were added, resulting in a molar ratio of MCPA:MAA:EGDMA = 1:15:7 [37]. The mixture was sonicated for 20 min and then purged with nitrogen for 5 min. The prepolymerization solution for the non-imprinted polymer (NIP) was prepared following the same procedure as for the MIP, except that no template (MCPA) was included.

### 2.3. Equipment

The experimental setups for testing both the POF-MIP sensors include a halogen lamp (HL-2000LL, Ocean Insight, Orlando, FL, USA) with an emission range of 360–1700 nm, used as a light source and spectrometers used as detectors. The spectrometers (FLAME-S-VIS-NIR-ES, Ocean Insight, Orlando, FL, USA) presented a detection range of 350–1023 nm and a 1.5 nm resolution, computed at the full width at half maximum (FWHM).

### 2.4. Model and Measuring Protocol for the Binding Tests

The POF-MIP and the POF-NIP sensor configurations were tested by dropping small volumes (50 µL for the SPR-POF-MIP and the SPR-POF-NIP, 10 µL for the POF-MIP and POF-NIP splitter sensors) of standard solutions at increasing MCPA concentrations over the sensitive area of the specific sensors, i.e., the MIP and the NIP layer for the plasmonic configurations and the micro-trench filled with the MIP or NIP in the case of the splitter sensors. The MCPA solutions were left to incubate with the MIP receptor for 10 min to allow the interaction of the analyte with the recognition cavities [41]. At the end of the incubation time, the sensitive area was washed three times with ultrapure water to remove every trace of MCPA non-specifically adsorbed on the polymer, following the procedure reported in [41]. The transmission spectra were then acquired with ultrapure water as the bulk solution. More specifically, for the plasmonic sensors, spectra were obtained by normalizing the transmitted spectra to the reference spectrum recorded in air, where the SPR condition is not satisfied [16]. On the other hand, the POF-MIP and the POF-NIP splitter sensors’ response (P_c_) was obtained by normalizing the propagated power at the direct output (P_d_) to the indirect output (P_i_), and considering it at a fixed wavelength, as described in [22,23]. For the SPR-POF-MIP and SPR-POF-NIP sensor configurations, the change in resonance wavelength relative to that of the blank solution (water free of MCPA) was monitored (Δλ = Δλ_c_ − Δλ_0_) at increasing MCPA concentrations [41]. Regarding the POF-MIP and POF-NIP splitter sensors, the normalized analytical signal at a specific concentration *c* (Y_c_) was achieved as the ratio between P_c_ and P_0_ (at a fixed wavelength), the latter calculated as the ratio between direct and indirect power measured at zero concentration (Y_c_ = P_c_/P_0_), as described in [22,23]. All the data were processed using MATLAB 2023a software.

The obtained experimental values were modeled by using the Hill equation, reported in (1), as follows:

(1) $$|\Delta S|={|S}_{C}-S_{0}|=|\Delta S_{max}|\cdot \left(\frac{c^{n}}{K^{n}+c^{n}}\right)$$

where Δ*S* is the resonance wavelength variation, Δλ, for the SPR-POF-MIP sensor chip and the normalized analytical signal, Y_c_, for the POF-MIP splitter sensor; $S_{C}$ and $S_{0}$ are the recorded signals, respectively, at c concentration and at zero concentration (solution without analyte); $\Delta S_{max}$ is the maximum value of ΔS obtained at the saturation of the MIP recognition sites, *n* and K are fitting constants. When *n* is considered to be equal to 1, the Hill fitting reported in (1) corresponds to the Langmuir one, K being the reciprocal of the affinity constant ($K_{aff}$) of the MIP binding sites for the target analyte. At low concentrations, when *c* is much lower than K, Equation (1) can be considered a linear function, and the slope ($\Delta S_{max}$/K) represents the so-called sensitivity at low concentrations. The limit of detection (LOD) can be calculated as 3.3 times the standard error of the intercept from the Hill fit (which can be considered not significantly different from the standard deviation of replicate blank measurements) divided by the sensitivity at low concentrations.

## 3. Sensor Systems

### 3.1. Surface Plasmon Resonance (SPR)–Plastic Optical Fiber (POF) Sensor Combined with A Molecularly Imprinted Polymer (MIP)

**Figure 1:**
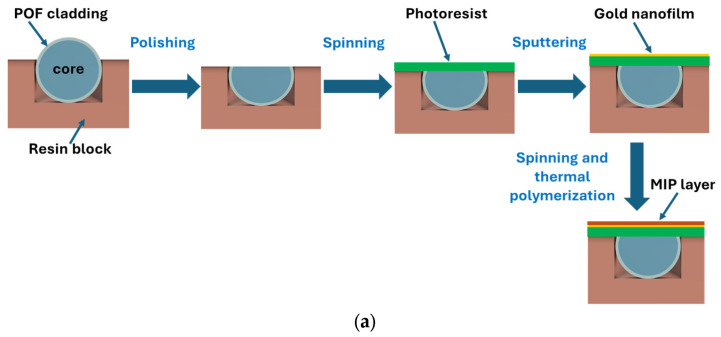

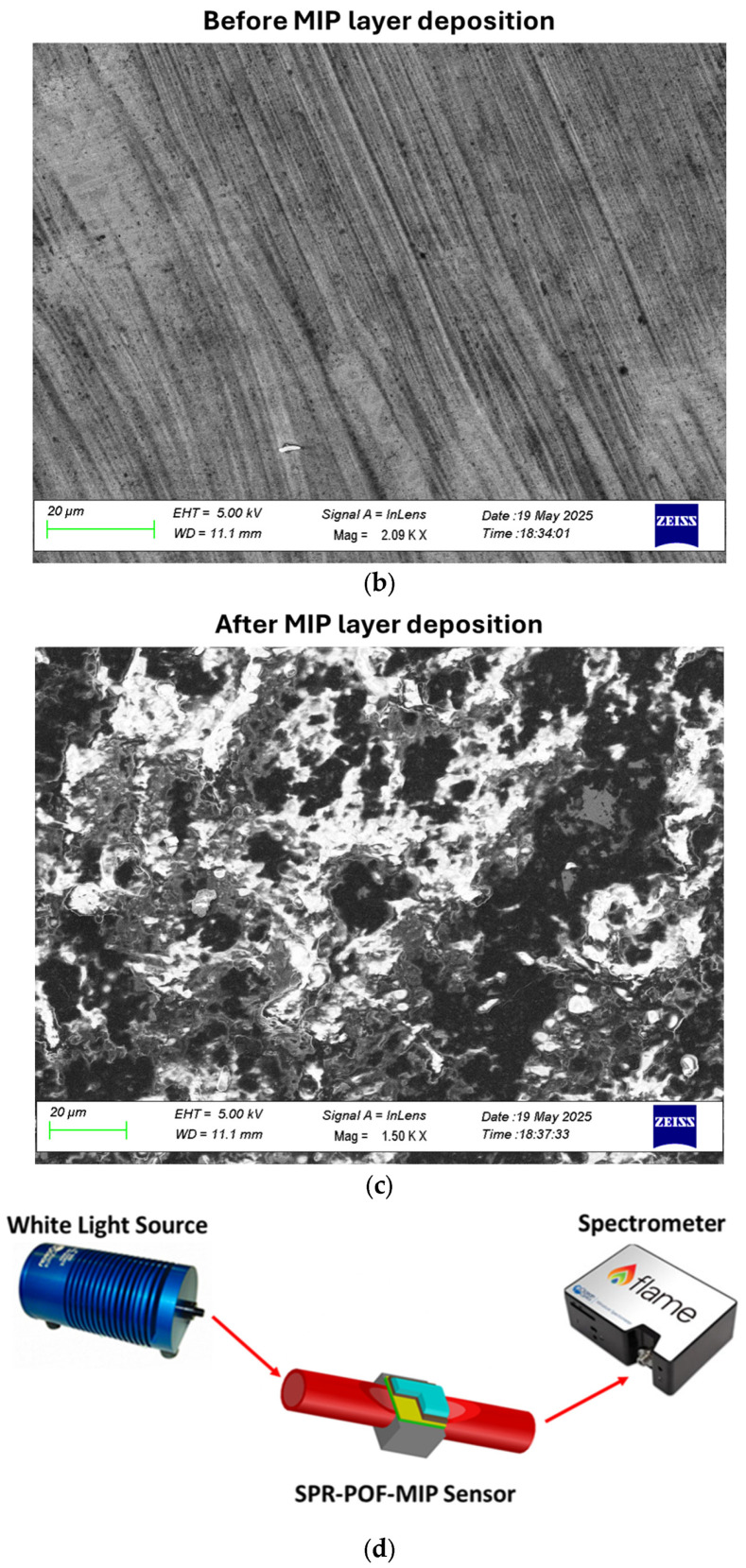
SPR-POF-MIP sensor: (**a**) schematic cross-section of sensor’s fabrication steps, and (**b**) scanning electron microscope image of the sensitive gold surface before the MIP layer deposition, (**c**) scanning electron microscope image of the sensitive surface after the MIP layer deposition, (**d**) outline of the implemented experimental setup, made up of a halogen lamp as a white light source and a spectrometer as a detector.

The plasmonic platform was fabricated following the conventional protocol described in [16]. Briefly, the SPR chip is based on a modified POF (PMMA core with a diameter of 980 µm and fluorinated polymer cladding of 10 µm), embedded in a trench (10 mm × 1 mm × 1 mm) of resin block, 10 mm long. In particular, the POF cladding and part of the core are removed using polishing paper with different grits (5 µm followed by 1 µm) to obtain the conventional D-shaped region [16]. Spinning and sputtering processes then follow first to create a layer of photoresist buffer (Microposit S1813, Milan, Italy, approximately 1 µm thick) and later to deposit a gold nanofilm (60 nm) on the sensitive surface of the exposed core [16]. Finally, the MIP layer deposition on the gold nanofilm has been achieved by exploiting a well-known strategy [41,42]. The plasmonic gold surface coating occurs directly over the flat sensitive area. Specifically, 40 µL of the MIP prepolymer mixture was spun onto the SPR-POF platform for 2 min at 1000 rpm and thermally polymerized in a thermo-static oven (at 70 °C overnight) [41,42]. The spinning step on the planar surface (achieved by polishing steps) is exploited to carry out uniform and thin MIP layers. The template and the free monomers were removed by washing steps with ethanol and rinsing the chip several times with ultrapure water [41,42]. Specifically, the uniformity of the 60 nm gold layer is ensured by the sputtering deposition process and by the small dimensions of the sputtering chamber (Safematic CCU-010, Zizers, Switzerland), which guarantee a homogeneous coating across the flat surface of the optical fiber. The MIP layer thickness, in the absence of a profilometer, was indirectly estimated from the sensor’s operating point and optical response, indicating a film thickness below 50 nm. The realized MIP layer and the gold nanofilm were investigated by SEM analysis, as reported in Figure 1. Specifically, Figure 1a schematizes the manufacturing steps for the SPR-POF-MIP sensor, while Figure 1b,c show the SEM images of the sensitive surface before and after MIP layer deposition, respectively. Finally, Figure 1c presents an outline of the experimental setup used. The SPR-POF-NIP sensor configuration was realized following the same protocol as the MIP-based configurations.

### 3.2. Optical Splitter Sensor Based on Plastic Optical Fibers (POFs) Combined with Molecularly Imprinted Polymers (MIPs)

**Figure 2:**
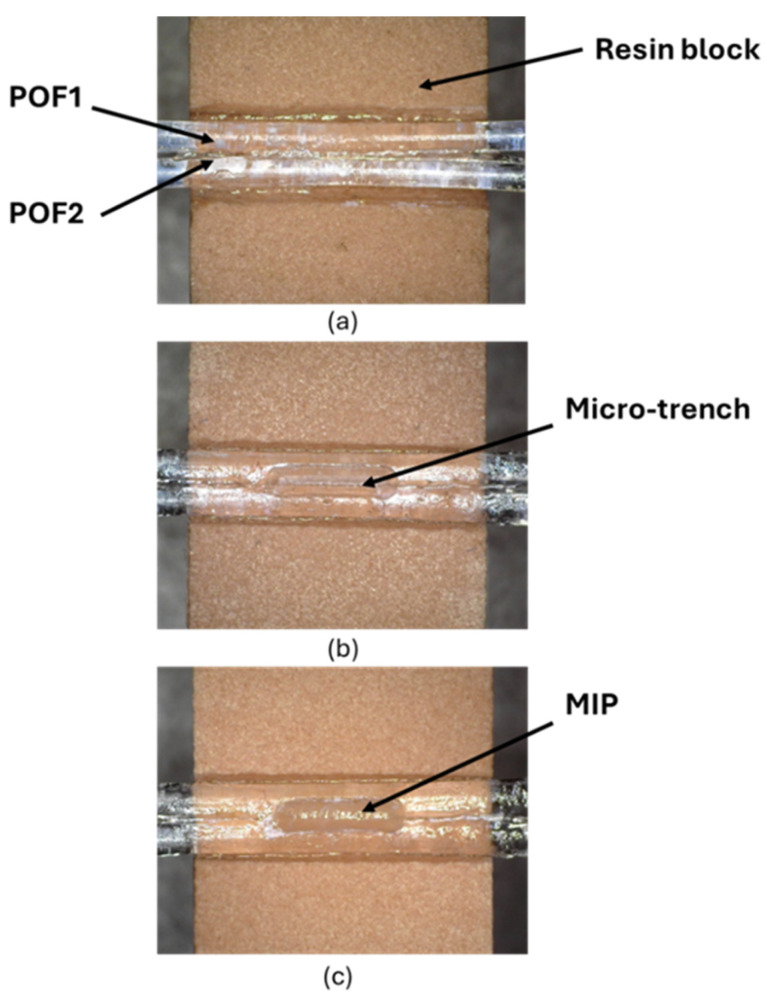
POF-MIP splitter sensor fabrication steps: (**a**) plastic optical fibers fixed into the resin block; (**b**) micro-trench made between the two coupled plastic optical fibers; (**c**) micro-trench filled with molecularly imprinted polymers.

**Figure 3:**
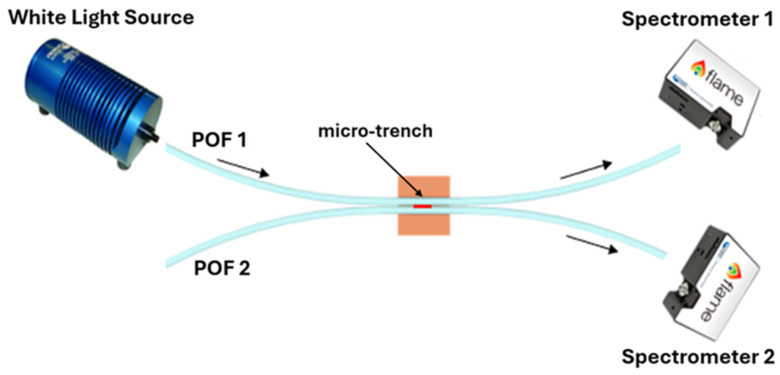
Outline of the experimental setup used to test the POF-based splitter sensors, consisting of a halogen lamp as a white light source and two spectrometers as detectors.

The splitter configuration was fabricated following the low-cost protocol described in [22,23]. Specifically, the realization process consisted of fixing two POFs in parallel mode into a trench (10 mm × 2 mm × 1 mm) of a resin block 10 mm long via a commercial glue (liquid cyanoacrylate, “Super Attak Loctite”). Then, a micro-trench (6000 μm (length) × 1000 μm (depth) × 400 μm (width)) is made among the two coupled POFs by a computerized numerical control (CNC) micro-milling machine. Lastly, the micro-trench is filled with 10 µL of the prepolymeric mixture, allowing the specific MIP to be obtained in the micro-area after polymerization and extraction [22,23]. In this case, the thickness of the MIP plays a different role than in the SPR method. The MIP thickness depends on the specific size of the micro-trench in the POFs, and its thickness is not critical with respect to the SPR technique. The realized MIP receptor was monitored using the digital microscope, as reported in Figure 2. Specifically, Figure 2 shows images of the splitter sensor acquired by the digital microscope (model Dino-Lite, manufactured by AnMo Electronics Corporation, New Taipei City, Taiwan) after each production step. To test the intensity-based sensor, a halogen lamp and two spectrometers were used to collect light from the POF directly connected to the light source (POF1) and from the indirect POF (POF2) [22,23], as outlined in Figure 3. The POF-NIP splitter configuration was realized following the same protocol as the MIP-based configurations.

## 4. Results and Discussion

Before starting with the dose–response curve experiments, the temporal evolution of the signal was monitored after each incubation of the MCPA solutions. Both sensors reached 95% of the final response within 8 ± 2 min, consistent with the adopted 10 min incubation time. Regeneration was evaluated by washing the surface with ethanol. Up to three regeneration cycles were achieved, after which partial loss of binding efficiency was observed.

**Figure 4:**
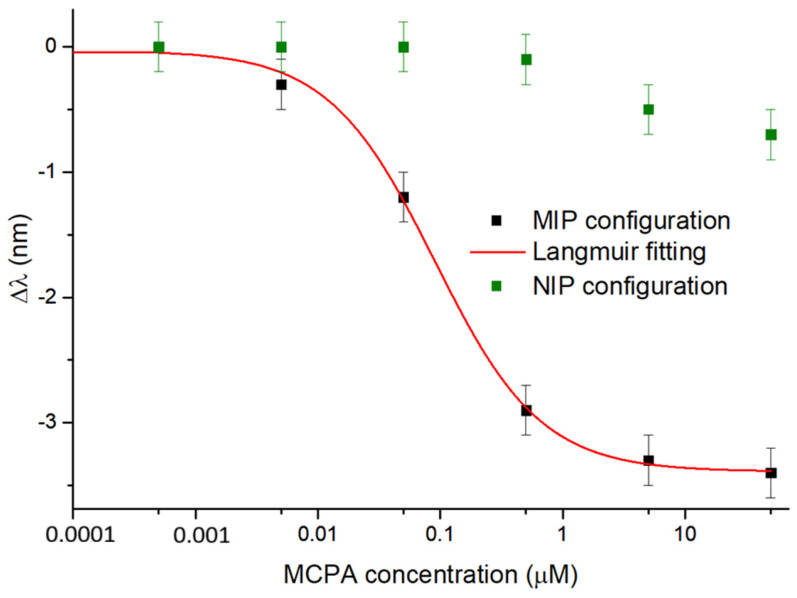
Experimental results of the binding tests performed on the SPR-POF-MIP and SPR-POF-NIP configurations at increasing MCPA concentrations, with the error bar (about 0.2 nm). The red curve is the dose–response curve of the sensor in a semilogarithmic scale achieved by the Langmuir fitting of the experimental data (black squares) by the MIP-based configuration. The green squares are the result values due to the experimental tests relative to the NIP-based configuration.

The experimental results obtained with the SPR-POF-MIP and SPR-POF-NIP sensor configurations, following the measurement procedure described in Section 2.4, are reported in Figure 4. In particular, Figure 4 shows the resonance wavelength changes concerning the blank, obtained by testing the sensors with different MCPA solutions (ranging from 0.5 nM to 50 µM). The dose–response curve relative to the MIP configuration was achieved by fitting the experimental data with (1) and considering n = 1. In Figure 4, the reported error bar corresponds to the maximum standard deviation observed when testing three similar sensor chips under the same conditions [41]. This value is equal to 0.2 nm. As shown in Figure 4, upon MIP–analyte interaction, the RI of the MIP layer decreases. This results in a shift in the resonance wavelength towards smaller values (blue-shift), similar to what is reported in the paper by Cennamo et al. [42], where the same SPR-POF probe was coupled with an MIP specific for perfluorinated compound detection. On the other hand, in the NIP configuration, no significant shift in the resonance wavelength was observed with increasing MCPA concentrations, compared to that recorded with the SPR-POF-MIP sensor. This confirms the sensor’s response is caused by the specific MCPA-MIP interaction.

In Table 1, the Hill fitting parameters obtained using OriginPro 9 software (OriginLab Corp., Northampton, MA, USA) with n = 1 are reported. The corresponding sensor’s performance parameters, in terms of the affinity constant, sensitivity at low concentrations, and limit of detection, are summarized in Table 2.

**Figure 5:**
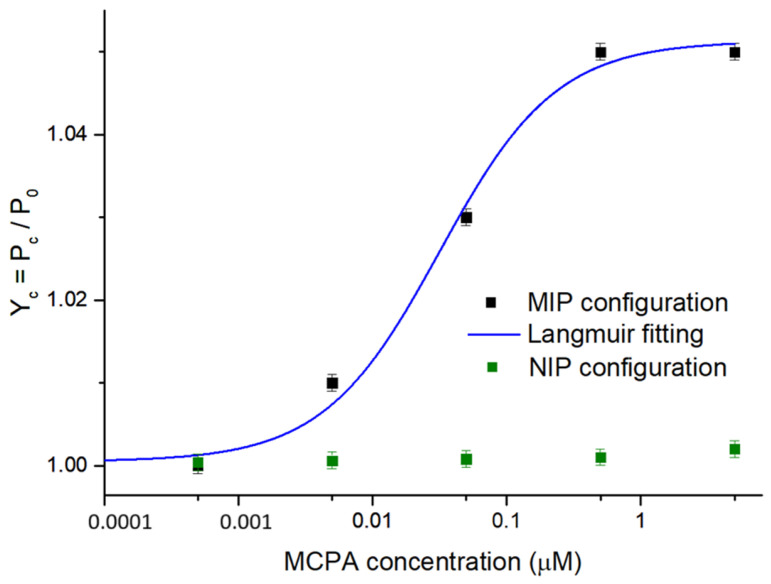
Experimental results of the binding tests performed on the POF-MIP and POF-NIP splitter configurations at increasing MCPA concentrations, with the error bar (about 0.001 a.u.). The blue curve is the dose–response curve in a semi-log scale obtained by the Langmuir fitting of the experimental values (black squares) from the MIP-based configuration. The green squares are the results of testing the NIP-based configuration.

The second developed MCPA sensor, i.e., the POF-MIP splitter sensor, was tested with increasing concentrations of MCPA (from 0.5 nM to 5 μM) to compare its performance with that attained by the SPR-POF-MIP sensor. In Figure 5, the dose–response curve relative to the MIP-based splitter configuration is reported together with the fitting of the experimental data via (1). Similarly to Figure 4, the error bar corresponds to the maximum standard deviation value observed by testing three similar sensor chips in the same conditions [22,23], which resulted in about 0.001 a.u. A control analysis was performed using the POF-NIP splitter sensor to demonstrate the specificity of the MIP binding sites towards the target analyte. As shown in Figure 5, the NIP-based configuration did not exhibit a significant response to increasing MCPA concentrations.

Regarding the splitter sensor, the normalized transmitted light intensity variation at a fixed wavelength (equal to 685 nm) was considered [22,23]. In fact, the MIP’s RI in the micro-trench changes upon binding to MCPA. Consequently, the MIP-MCPA interaction results in a change in the waveguide coupling properties in terms of normalized transmitted light intensity, as explained by Tavoletta et al. and Cennamo et al. [22,23]. More specifically, the monitored Y_c_ signal, achieved as indicated in Section 2.4, increases as the concentration of MCPA tested increases, as shown in Figure 5. Table 3 shows the Hill fitting parameters, while Table 4 summarizes the chemical parameters achieved by the POF-MIP splitter.

It should be stressed that, for both SPR-POF-MIP and POF-MIP splitter sensors, the Hill model was applied with *n* = 1 to represent a Langmuir-type isotherm, as the experimental binding behavior was found to be well described by a single-site model.

**Figure 6:**
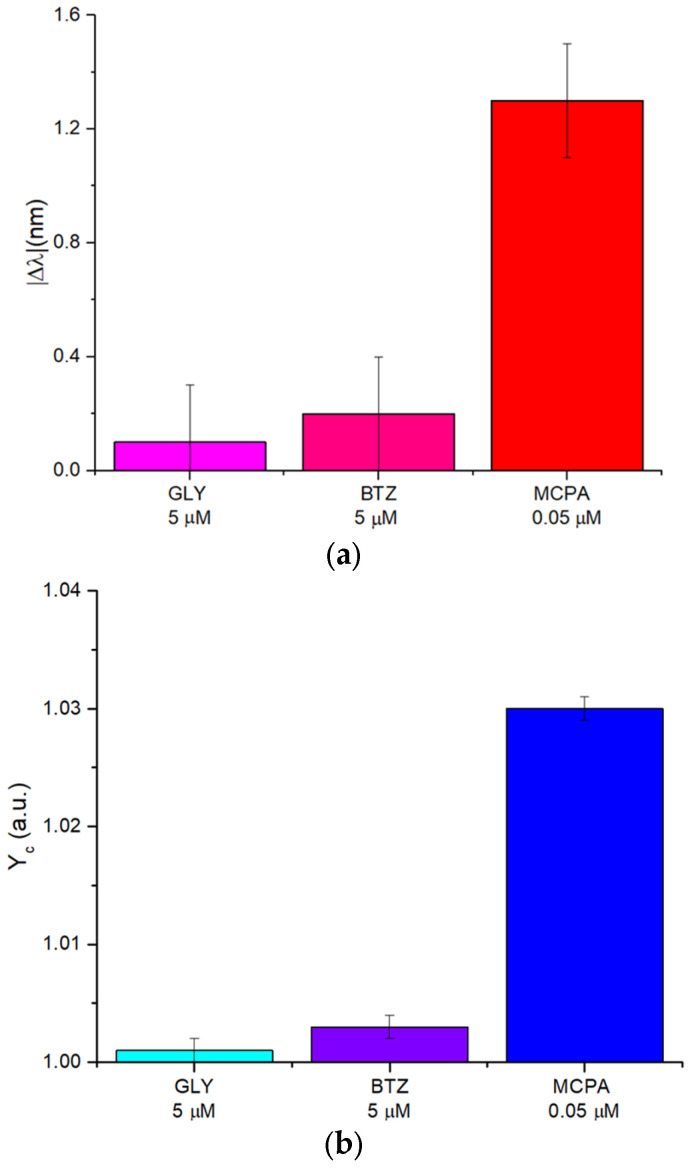
POF-MIP sensors selectivity tests: (**a**) comparison of the signal |Δλ| [nm] produced by interfering substances (glyphosate and bentazone) concentrated at 5 µM, compared to that recorded in the presence of the target analyte (MCPA) at 0.05 µM. (**b**) Comparison of the signal Y_c_ [a.u.] produced by interfering substances (glyphosate and bentazone) concentrated at 5 µM, compared to that recorded in the presence of the target analyte (MCPA) at 0.05 µM.

To evaluate the synthetic receptor’s selectivity, both POF-MIP-based sensor configurations were tested with potential interfering substances, including the herbicides glyphosate (GLY) and bentazone (BTZ). Selectivity studies with structural analogues (Mecroprop, Dichloroprop, and 2,4-D Pestanal) have already been carried out in [37], where the same MIP was combined with an electrochemical probe. As reported in those tests, the polymer is selective for the entire class of phenoxy herbicides with structures similar to the template MCPA. Specifically, the SPR-POF-MIP sensor (Figure 6a) and the POF-MIP splitter sensor (Figure 6b) were tested with high concentrations of GLY and BTZ (about 5 μM). Still, the responses in both sensor configurations were not significantly different from those recorded in the presence of the target analyte (MCPA), as shown in Figure 6, even though the tested MCPA concentration (about 0.05 μM) was two orders of magnitude lower than the concentration of the interferents. Therefore, these analyses demonstrated the high selectivity of both POF-MIP sensor systems.

Moreover, for both the developed optical–chemical sensors, the imprinting factor (IF) was evaluated. In particular, the IF is a key parameter in molecular imprinting materials and is calculated by comparing the optical responses at the same analyte concentration obtained with the imprinted material (MIP) and the non-imprinted material (NIP) configurations. For a specific recognition, the IF should be higher than 1 [43]. Table 5 reports the optical–chemical sensors’ IFs calculated at three different MCPA concentrations (low, medium, and high) for both MIP and NIP configurations. Specifically, the IF is achieved from the ratio between the signals obtained with the MIP configuration and the NIP ones, i.e., |Δλ| for the SPR-POF configurations and ΔY_c_ for the splitter sensors, where ΔY_MIP_ and ΔY_NIP_ are Y_MIP_ and Y_NIP_, respectively, each subtracted by one.

Finally, to show the sensor systems’ application in a real scenario, the experimental results obtained by testing both the polymer-based sensors for MCPA detection in real matrices are reported in Table 6. Specifically, distilled and drinking water samples spiked with MCPA 50 nM and 5 nM were tested on the SPR-POF-MIP sensor and the POF-MIP splitter sensor, respectively. As shown in Table 6, the recovery percentages obtained are consistent with acceptable standards [44]. A slight effect of signal suppression due to the sample matrix was observed for tap water, but in any case, recovery values greater than 80% are adequate for quantitative analysis.

**Figure 7:**
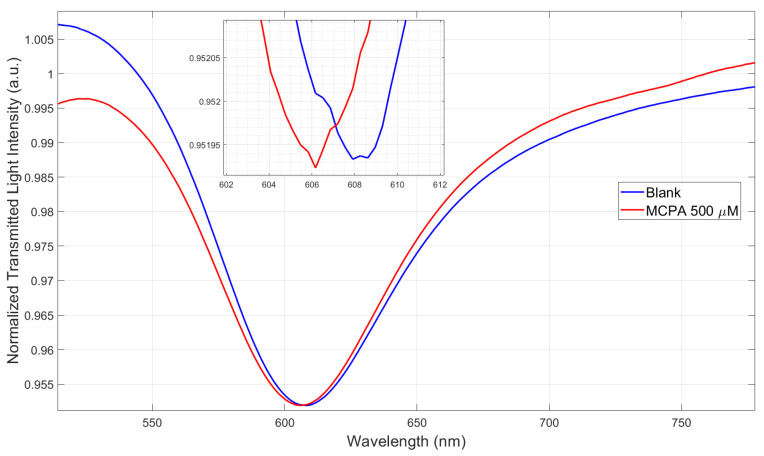
Plasmonic spectra acquired via the SPR-POF probe (bare surface) in the presence of the blank solution (ultrapure water) and ultrapure water with 500 μM of MCPA.

Both tested POF-based sensors show that the MIP’s RI decreases upon binding to MCPA. Specifically, the MIP-MCPA binding causes a decrease in the receptor layer RI deposited on the SPR-POF platform, resulting in a blue-shift in the resonance wavelength (Figure 4), similar to what is observed with the same SPR-POF probe coupled with an MIP specific for perfluorinated compound detection [42]. As an additional confirmation, the increase in the intensity signal (Yc) observed when testing the splitter sensor with increasing MCPA concentrations (Figure 5) is also consistent. In fact, the splitter-based configuration tested with an MIP for 2-FAL detection produced a decrease in Yc, as this MIP’s RI increased with increasing 2-FAL concentrations [22]. As a further control, we compared the SPR spectra of an SPR-POF probe (bare surface without MIP layer) in ultrapure water vs. ultrapure water added with a high concentration of MCPA, 500 µM of MCPA (Figure 7), confirming a blue-shift when high MCPA concentrations were added to the medium, directly showing the RI reduction in the bulk solution. In fact, as shown in the SPR spectra variation reported in Figure 7, for this type of SPR chip, the shift towards lower wavelengths is due to a decrease in the RI at the interface with the SPR gold surface [16]. Therefore, this result is consistent with the binding tests performed via the POF-MIP sensors (Figure 4 and Figure 5), where an increasing MCPA concentration in the MIP medium decreases the MIP’s RI.

In our previous works, where the SPR-POF platforms were combined with MIPs for different analytes of interest, such as 2-FAL and dibenzyl disulfide (DBDS) [41,45], the receptor–analyte interaction increased the MIP layer RI, thus producing a shift in the wavelength towards higher values (red-shift), as the tested analyte concentration increased. In the splitter sensors exploiting the same synthetic receptors [22,23], as the analyte concentration changed, the MIP RI in the micro-trench changed (increased), and this resulted in a decrease in the signal Y_c_. In the case of the SPR-POF-MIP sensor for MCPA instead, the interaction with the analyte results in a decrease in the receptor layer RI, resulting in a blue-shift in the resonance wavelength (Figure 4). Thus, the increase in intensity signal (Y_c_), observed when testing the splitter sensor with increasing MCPA concentrations (Figure 5), is consistent. A comparative analysis, in terms of sensor responses of different optical–chemical chips, both plasmonic and intensity-based, is shown in Table 7 to emphasize the consistency of the results obtained here.

The two proposed optical–chemical sensors based on the same MIP showed similar chemical performance for MCPA detection, even though they used different optical sensing principles (plasmonic phenomena and POFs coupling via MIPs in intensity-based configurations). The obtained LOD of 3 nM is lower than that achieved with the electrochemical platform functionalized with the same MIP (about 13 nM) [37], demonstrating the greater sensitivity of the here-proposed optical sensors. It is also worth noting that intensity-based systems, such as splitter sensors, perform similarly to POF-based plasmonic platforms combined with an MIP layer, as demonstrated in [22,23]. Also in this work, the results of the dose–response curves, performed on both the proposed polymer-based sensors, show fully comparable performance for MCPA detection, as summarized in Table 8. In particular, the results reported in Table 8 demonstrated the possibility of exploring intensity-based sensor systems for the MCPA monitoring in water, rather than plasmonic ones, which are more expensive to implement.

To further demonstrate the good performance of the here-proposed devices, Table 9 summarizes the state of the art of MCPA sensors (optical and electrochemical). As shown in Table 9, electrochemical sensors, such as the potentiometric one proposed by Zanoni et al. based on the same MIP here proposed [37], or the voltametric method using activated glassy carbon described by Yu et al. [46], and also two optical methods, i.e., the SERS one based on 2D silver nanodendrites functionalized with cyclodextrin proposed by Daly et al. [47], and the quartz microbalance-based sensor modified with a fibrous-like molecularly imprinted polymer film described by Si et al. [48], are the least sensitive, with the highest LODs. In contrast, optical sensors based on fluorescent carbon dots presented by Haddadou et al. [49], and those proposed in the present study, are promising for the detection of MCPA at trace levels. Moreover, a recent study [50] reported two optical sensors: a conventional SPR-POF-MIP sensor and an MIP core configuration, both functionalized with the same MIP used here, enabling an ultra-wide MCPA detection range. While the plasmonic sensor exhibited performance comparable to that obtained in the present work, the MIP core configuration exploited different affinity sites of the synthetic receptor, resulting in a different detection range and LOD approximately two orders of magnitude lower, as reported in Table 9.

In this work, the POF-MIP splitter sensor demonstrated chemical performance equivalent to that of the SPR-POF-MIP sensor, in terms of the LOD and detection range for the MCPA monitoring. Therefore, the developed splitter sensor could be combined with the MIP core configuration to achieve ultra-wide detection of this herbicide, instead of using the more expensive SPR-POF probe, providing a low-cost alternative system that does not exploit plasmonic phenomena. Moreover, although the EU Drinking Water Directive specifies a limit of 0.1 μg/L (0.5 nM) for MCPA, there is less uniformity among EU member states and associated countries regarding Environmental Quality Standards (EQSs). For instance, the annual average EQS values for MCPA reported across EU countries vary considerably, ranging from 0.01 to 1.6 μg/L (from 0.05 to 8 nM) [51]. Therefore, the splitter sensor coupled with the MIP core configuration could be sufficiently sensitive to detect MCPA within the EQS range, as is now only possible with gold-standard methods such as GC-MS and LC-MS [52].

## 5. Conclusions

In this work, in order to detect the herbicide MCPA in water samples, two optical–chemical sensors that combine POFs and MIPs were developed. In particular, the same MIP receptor was integrated into two different optical configurations: an SPR-POF probe and a POF-based splitter platform, allowing the evaluation of different optical phenomena for the same target molecule detection with great affinity and selectivity. The experimental results demonstrated that both sensors achieved comparable LODs around 3 nM and similar detection ranges (between 3 nM and 500 nM), significantly outperforming an electrochemical sensor based on the same MIP and other state-of-the-art MCPA sensors. In addition, the usability of both sensors in real-world scenarios was assessed by testing them with real samples (i.e., distilled water and tap water), yielding good recovery values (82–116%). Among the two configurations, the splitter-based sensor offers significant advantages over the SPR-POF. In fact, for the splitter-based chemical sensor, no gold deposition step is required, thereby reducing both the fabrication costs and complexity.

These findings highlight the potential of intensity-based POF-MIP sensors as reliable, low-cost, and efficient alternatives to traditional plasmonic devices for monitoring environmental contaminants. Moreover, the obtained results allow the splitter system to be combined with other more sensitive configurations, such as the MIP core sensor reported in [50], to obtain an ultra-wide detection range for the MCPA, instead of using the more expensive SPR-POF probe [50], providing a low-cost alternative system that does not explore plasmonic phenomena. Future studies could further explore improvements to the splitter sensor configuration and its integration with IoT technologies for real-time, remote monitoring of pollutants in real-world scenarios.

## Figures and Tables

**Table 1:** Hill fitting parameters obtained for the MCPA detection via the SPR-POF-MIP sensor.

Δλ_0_ [nm]		Δλ_max_ [nm]		*K* [μM]		*n*		Statistics
Value	St. err.	Value	St. err.	Value	St. err.	Value	St. err.	R^2^
−0.03	0.04	−3.39	0.04	0.089	0.008	1	0	0.998

**Table 2:** Chemical parameters for the MCPA detection via SPR-POF-MIP sensor.

Parameters	Value
Affinity constant [μM]^−1^	11.14
Sensitivity at low concentration [nm/μM]	37.44
Limit of detection [μM]	0.003

**Table 3:** Hill fitting parameters obtained for the MCPA detection via the POF-MIP splitter sensor.

ΔY_0_ [a.u.]		ΔY_max_ [a.u.]		*K* [μM]		n		Statistics
Value	St. err.	Value	St. err.	Value	St. err.	Value	St. err.	R^2^
1.000	0.002	1.05	0.02	0.032	0.007	1	0	0.991

**Table 4:** Chemical parameters for the MCPA detection via POF-MIP splitter sensor.

Parameters	Value
Affinity constant [μM]^−1^	31.5
Sensitivity at low concentration [a.u./μM]	1.6
Limit of detection [μM]	0.003

**Table 5:** Evaluation of the IFs for both the optical–chemical sensors at three different MCPA concentrations.

MCPA Concentration	SPR-POF Sensors		IF	Splitter Sensors		IF
	|Δλ_MIP_|[nm]	|Δλ_NIP_|[nm]		ΔY_MIP_ [a.u.]	ΔY_NIP_ [a.u.]	
5 nM	0.3	0.2	1.5	0.01	0.001	10
50 nM	1.2	0.2	6	0.03	0.001	30
500 nM	2.9	0.2	14.5	0.05	0.001	50

Legend: 4-chloro-2-methylphenoxyacetic acid (MCPA); surface plasmon resonance (SPR); plastic optical fiber (POF); molecularly imprinted polymer (MIP); non-imprinted polymer (NIP).

**Table 6:** Recovery test in distilled and tap water samples.

Sensor	Sample	MCPA Added [nM]	MCPA Found [nM]	Recovery [%]
SPR-POF-MIP	distilled water	50.0	50.3	101
	tap water	50.0	42.5	85
POF-MIP splitter	distilled water	5.0	5.8	116
	tap water	5.0	4.1	82

Legend: Surface plasmon resonance (SPR); plastic optical fiber (POF); molecularly imprinted polymer (MIP); 4-chloro-2-methylphenoxyacetic acid (MCPA).

**Table 7:** Comparative analysis between optical–chemical sensors for several analytes of interest.

Optical–Chemical Sensors	Analyte	Behavior of the MIP-Based Sensor	Ref.
SPR-POF-MIP	DBDS	Red-shift	Cennamo et al. [45]
POF-MIP splitter	DBDS	Intensity decrease	Cennamo et al. [23]
SPR-POF-MIP	2-FAL	Red-shift	Pesavento et al. [41]
POF-MIP splitter	2-FAL	Intensity decrease	Tavoletta et al. [22]
SPR-POF-MIP	MCPA	Blue-shift	[This work]
POF-MIP splitter	MCPA	Intensity increase	[This work]

Legend: Surface plasmon resonance (SPR); plastic optical fiber (POF); molecularly imprinted polymer (MIP); dibenzyl disulfide (DBDS); furfural (2-FAL); 4-chloro-2-methylphenoxyacetic acid (MCPA).

**Table 8:** Performance comparison of MCPA POF-MIP sensors.

POF-MIP Sensors	Limit of Detection	Affinity Constant
SPR-POF-MIP	3 nM	11.14 [μM]^−1^
POF-MIP splitter	3 nM	31.5 [μM]^−1^

Legend: Surface plasmon resonance (SPR); plastic optical fiber (POF); molecularly imprinted polymer (MIP).

**Table 9:** MCPA sensors and methods: comparative analysis.

MCPA Sensors	Sensing Principle	Matrix	LOD	Ref.
Potentiometric MIP-modified screen-printed cell	Electrochemical	Phosphate buffer	13 nM	Zanoni et al. [37]
Activated glassy carbon electrode	Electrochemical	Phosphate buffer	8 nM	Yu et al. [46]
2D silver nanodendrites functionalized with cyclodextrin for SERS sensing	Optical	Water	10^6^ nM	Daly et al. [47]
(F-MIP)-based QCM sensor	Optical	Water	200 nM	Si et al. [48]
Fluorescent chitosan hydrogels based on biomass-derived carbon dots	Optical	Water	4 nM	Haddadou et al. [49]
SPR-POF-MIP sensor	Optical	Water	3 nM	[This work]
MIP core sensor	Optical	Water	0.08 nM	Tavoletta et al. [50]
POF-MIP splitter	Optical	Water	3 nM	[This work]

Legend: 4-chloro-2-methylphenoxyacetic acid (MCPA); molecularly imprinted polymer (MIP); fibrous-like MIP (F-MIP); quartz crystal microbalance (QCM); surface plasmon resonance (SPR); plastic optical fiber (POF).

## Data Availability

The original contributions presented in this study are included in the article. Further inquiries can be directed to the corresponding authors.
